# Derangement of Metabolic and Lysosomal Gene Profiles in Response to Dexamethasone Treatment in Sarcoidosis

**DOI:** 10.3389/fimmu.2020.00779

**Published:** 2020-05-12

**Authors:** Jaya Talreja, Christian Bauerfeld, Edward Sendler, Roger Pique-Regi, Francesca Luca, Lobelia Samavati

**Affiliations:** ^1^Division of Pulmonary, Critical Care and Sleep Medicine, Department of Internal Medicine, School of Medicine and Detroit Medical Center, Wayne State University, Detroit, MI, United States; ^2^Division of Critical Care, Department of Pediatrics, School of Medicine and Detroit Medical Center, Wayne State University, Detroit, MI, United States; ^3^Center for Molecular Medicine and Genetics, School of Medicine, Wayne State University, Detroit, MI, United States; ^4^Department of Obstetrics and Gynecology, School of Medicine, Wayne State University, Detroit, MI, United States

**Keywords:** dexamethasone, corticosteroids, gene expression, sarcoidosis, alveolar macrophages, monocytes, RNA-seq

## Abstract

Glucocorticoids (GCs) play a central role in modulation of inflammation in various diseases, including respiratory diseases such as sarcoidosis. Surprisingly, the specific anti-inflammatory effects of GCs on different myeloid cells especially in macrophages remain poorly understood. Sarcoidosis is a systemic granulomatous disease of unknown etiology that occurs worldwide and is characterized by granuloma formation in different organs. Alveolar macrophages play a role in sarcoidosis granuloma formation and progressive lung disease. The goal of the present study is to identify the effect of GCs on transcriptomic profiles and the cellular pathways in sarcoidosis alveolar macrophages and their corresponding blood myeloid cells. We determined and compared the whole transcriptional signatures of alveolar macrophages from sarcoidosis patients and blood CD14^+^ monocytes of the same subjects in response to *in vitro* treatment with dexamethasone (DEX) via RNA-sequencing. In response to DEX, we identified 2,834 genes that were differentially expressed in AM. Predominant pathways affected were as following: metabolic pathway (FDR = 4.1 × 10^−10^), lysosome (FDR = 6.3 × 10^−9^), phagosome (FDR = 3.9 × 10^−5^). The DEX effect on AMs is associated with metabolic derangements involving glycolysis, oxidative phosphorylation and lipid metabolisms. In contrast, the top impacted pathways in response to DEX treatment in blood CD14^+^ monocytes were as following; cytokine-cytokine receptor interaction (FDR = 6 × 10^−6^) and transcriptional misregulation in cancer (FDR = 1 × 10^−4^). Pathways similarly affected in both cell types were genes involved in lysosomes, cytoskeleton and transcriptional misregulation in cancer. These data suggest that the different effects of DEX on AMs and peripheral blood monocytes are partly dictated by lineage specific transcriptional programs and their physiological functions.

## Introduction

Pulmonary sarcoidosis is characterized by granuloma formation in the lungs. Pulmonary involvement is the leading cause of morbidity and mortality in sarcoidosis patients in the US, while other organ involvements including eyes, central nervous system, cardiac, and skin may lead to severe functional impairments (1, 2). Lungs are constantly exposed to toxins and microbial products and lung macrophages play important roles in the maintenance of immunological homeostasis and are the cornerstone of granuloma formation (1, 3, 4).

Due to their immune-modulatory actions, glucocorticoids (GCs) are one of the most widely prescribed drugs for the treatment of inflammatory and immune mediated disorders, including sarcoidosis (5). The long-term use of GCs is limited due to profound derangements of glucose metabolism, mineral homeostasis, amino acid metabolism, cognition and effects on the cardiovascular system (6). Several studies questioned the long-term benefits of GCs treatment in several respiratory diseases, including chronic obstructive lung disease (COPD). In idiopathic pulmonary fibrosis GCs treatment has been associated with worsening prognosis (7–9). Similarly, the long term benefits of GC treatments in sarcoidosis is unclear (7). While GCs suppress inflammation by decreasing lymphocyte activation, proliferation, and survival, there is a knowledge gap in how GCs modulate the immune response of human myeloid cells and tissue macrophages, especially in sarcoidosis. Recent studies highlighted that GCs have little effect in controlling inflammation in macrophage-dominated diseases (8, 10).

Because AMs originated ontologically from yolk sac and adapt to maintain pulmonary hemostasis and clearance, the effect of GCs on AMs may be different as compared to myeloid derived macrophages and monocytes (11). Additionally, in sarcoidosis the response to GCs treatment is partly dictated by organ involvement. This posits to question if tissue macrophages, especially AMs as compared to monocytes, are less susceptible to GCs treatment. Dissecting the effect of GCs treatment on cellular responses of two different macrophage lineages may uncover important molecular pathways to discriminate beneficial from detrimental effects of this therapy.

The goal of this study was to identify the differential transcriptional signatures and signaling pathways affected by corticosteroids in two relevant macrophage lineages (AMs and peripheral monocytes) in sarcoidosis. We performed transcriptomic analysis of isolated AMs and CD14^+^ monocytes from the same sarcoidosis patients in response to *ex-vivo* treatment with DEX. We identified common as well as unique pathways affected by GCs in sarcoidosis AMs and monocytes.

## Methods

### Study Subjects

Sarcoidosis diagnosis was based on the ATS/ERS/WASOG statement (12). The enrollment criteria for sarcoidosis patient were as previously described (13–15). A total of 10 patients with sarcoidosis participated in this study.

### Isolation of AMs From BAL

AMs were isolated from BAL fluid as previously described (14, 15). Viability of AMs was routinely about 98%. Immunofluorescent staining with CD68 antibody confirmed >99% purity.

### Isolation and Purification of CD14^+^ Monocytes

CD14^+^ monocytes were purified from PBMCs by using the MACS monocyte isolation kit (Miltenyl Biotech, San Diego, CA) according to the manufacturer's instructions (13, 14). The purity of enriched monocytes was evaluated by flow cytometry using fluorochrome-conjugated CD14 antibody; the purity of monocytes was >98%.

### mRNA Isolation

Sarcoidosis AMs or monocytes were cultured in the presence of DEX (100 ng/mL) or vehicle for 16 h. After 16 h incubation, mRNA was isolated from purified AMs and monocytes using the Dynabeads mRNA Direct Kit (Ambion) as described earlier (13).

### RNA-seq Library Preparation and Sequencing

RNA-seq libraries were prepared using the NEBNext ultradirectional library preparation protocol (New England BioLabs, Ipswich, MA) as described earlier (13). RNA-seq library quality was assessed using an Agilent Bioanalyzer. Individually barcoded RNA-seq libraries were pooled in equimolar quantities. A pooled library of 40 samples: untreated sarcoidosis AMs (*n* = 10), DEX-treated sarcoidosis AMs (*n* = 10), untreated sarcoidosis monocytes (*n* = 10), and DEX-treated sarcoidosis monocytes (*n* = 10) was sequenced on the Illumina Next-Seq 500 (75 cycles, PE).

### RNA-seq Data Analysis, Differential Gene Expression, and Canonical Pathway Analysis

RNA-seq data were analyzed using the Illumina Basespace RNA express application. The sequencing reads were aligned to the reference human genome hg19 using STAR aligner and differentially expressed (DE) genes (FDR < 5%) were identified with the DEseq2 analysis tool (16, 17). Enrichment of cellular pathway and gene ontology category was calculated using iPathwayGuidetool and Gene Trail 2 Over-Representation Analysis (ORA) and comparing the list of DE genes with background expressed genes. Pathway enrichment was determined at FDR < 5%, using Benjamini and Hochberg procedure to control for multiple testing (18).

## Results

### Effect of Dexamethasone on the Transcriptional Signature of Sarcoidosis AMs

**Figure 1:**
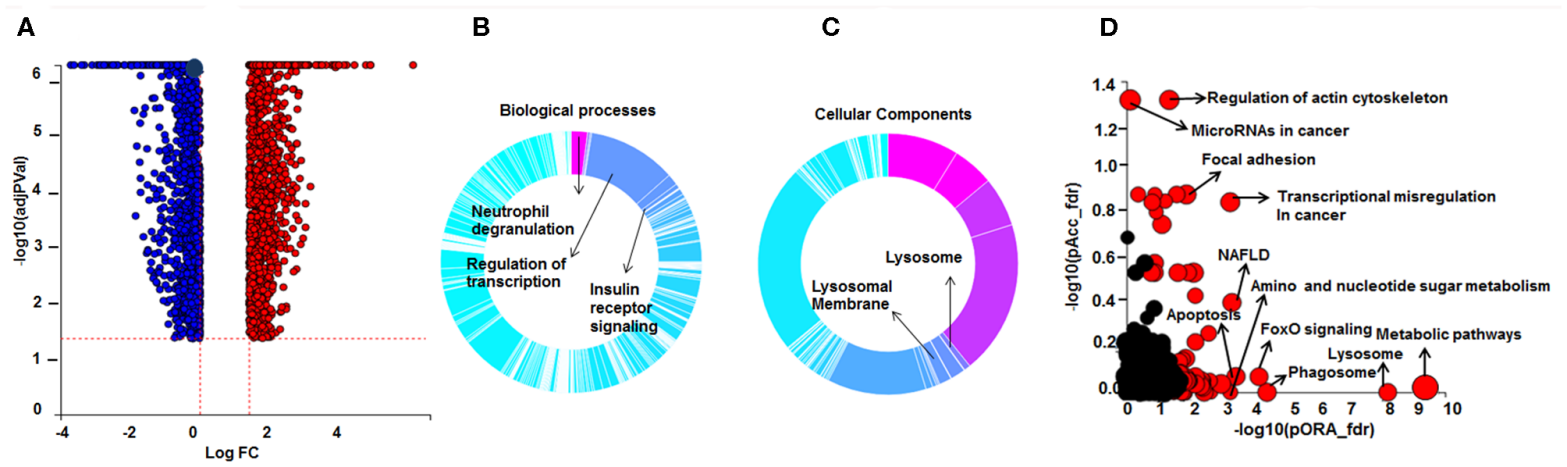
Differential gene expression and Gene Ontology (GO) processes and significant pathways between DEX-treated compared to untreated sarcoid AMs. **(A)** Volcano plot: 2,834 significant DE genes analyzed by DEseq2 analysis tool are represented in terms of their measured expression fold change (log_2_FC) on the x-axis and the significance of the change (–log_10_ adj *p*-value) on the y-axis. The dotted lines represent the thresholds used to select the DE genes: log_2_-fold change (>0.6) and significance (FDR < 5%). The up-regulated genes (positive log_2_FC) are shown in red, while the down-regulated (negative log_2_FC) genes are shown in blue. **(B,C)** Gene Ontology (GO) analysis of DE genes. To assess the enrichment of GO terms Elim pruning method was used. GO analysis identified top **(B)** biological processes and **(C)** cellular components. **(D)** Pathway analysis on the DE genes was performed considering a threshold of log_2_ FC > 0.6 and FDR < 5% using iPathwayGuide analysis tool that uses two types of evidence: the over-representation on the horizontal axis (pORA) and the perturbation on the vertical axis (pAcc). Significant pathways (FDR < 5%) are shown in red, whereas non-significant are in black. The size of the circle is proportional to the number of genes in that pathway.

Using CD68 antibody as macrophage marker, the purity of isolated AMs was >99% (Figure S1). The subject demographics are displayed in Table 1. All subjects were non-smokers, and none were on corticosteroid or other immune-suppressive medication. Isolated AMs were cultured either in the presence of DEX (100 ng/mL) for 16 h or vehicle. RNA-seq libraries were prepared from DEX treated AMs (*n* = 10) and untreated AMs (*n* = 10) from the same subjects serving as controls. RNA-seq libraries were sequenced on one lane of the Illumina Next-Seq 500 as described previously (13). Using DeSeq2, we identified 2834 DE genes (log_2_-fold change (FC) > 0.6 and FDR < 5%) between DEX-treated vs. untreated AMs from same sarcoidosis patients (Figure 1A). The Gene ontology (GO) enrichment analysis of DE genes (FDR < 5%) showed that the significant biological processes impacted after DEX treatment were neutrophil degranulation (FDR = 1.7 × 10^−7^), regulation of transcription (FDR = 3.3 × 10^−4^), and insulin receptor signaling (FDR = 4.1 × 10^−4^) (Figure 1B). Most genes involved in neutrophil degranulation were downregulated. CD14 is a general marker for myeloid cells (macrophages and monocytes), but it has been shown that activated neutrophil exhibit surface CD14 (19). The analysis of biological function enrichment showed lysosome and lysosomal membrane as significant cellular components affected by DEX treatment (Figure 1C). Table 2 summarizes the most up and down regulated genes in response to DEX treatment in sarcoidosis AMs. Increased expression of several of these genes has been reported in THP-1 cell lines or monocyte-derived dendritic cells in response to DEX treatment (20–22). We identified that DEX-treatment induced the expression of two novel genes; Ring finger protein (RNF) 175 and amine oxidase copper containing 2 (AOC2). Other upregulated genes were formyl peptide receptor 1 (FPR1) (log_2_ FC = 2.6, FDR = 1.8 × 10^−7^) and Vanin 1 (VNN1) (log_2_ FC = 2.0, FDR = 7.1 × 10^−7^). FPR1 is a member of the G-protein coupled receptor 1 family encoding protein (21), which mediates the response of phagocytic cells to microorganisms. Vanin 1 belongs to vanin gene family and has pantetheinase activity and participates in hematopoietic cell trafficking (23). Similarly, complement factor properdin (CFP) (log_2_ FC = 1.3, FDR = 7.4 × 10^−5^) was upregulated. This gene positively regulates the alternative complement pathway. DEX-treatment downregulated several small nucleolar RNA box genes, solute carrier families and integrin genes in sarcoid AMs.

**Table 1:** Subject demographics.

**Characteristic**	**Patients**
Age (year)	46.11 ± 14.4
BMI	29 ± 10.4
Gender, *N* (%)	
Female	5 (50)
Male	5 (50)
Self-reported Race, N (%)	
African American	10(100)
White	0 (0)
CXR stage, *N* (%)	
0	0 (0)
1	0 (0)
2	6 (60)
3	4 (40)
Organ Involvements, *N* (%)	
Neuro-ophthalmologic	2 (10)
Lung	10 (100)
Skin	4 (10)
Multiorgans	15 (75)
PPD	Negative
AFB/culture	Negative
IGRA	Negative

*BMI, body mass index; CXR, chest X-ray; PPD, purified protein derivative; AFB, acid-fast bacilli; IGRA, interferon gamma release assay*.

**Table 2:** Top up and down-regulated regulated genes in AMs in response to dexamethasone treatment.

**Gene symbol**	**Gene name**	**Type**	**log_**2**_ fold change**	**FDR *p*-value**
pkp2	Plakophilin 2	Cytoskeleton	4.5	7.87E-19
ABLIM3	Actin binding LIM protein family member 3	Cytoskeleton	3.5	1.23E-13
SRPX	Sushi repeat containing protein X-linked	Peroxiredoxin-like domain	3.5	4.03E-10
TSC22D3	TSC22 domain family member 3	Leucine zipper protein	3.4	5.13E-26
METTL7A	Methyltransferase like 7A	Methyltransferase	3.1	6.28E-14
MFGE8	Milk fat globule-EGF factor 8 protein	Membrane glycoprotein	2.9	6.59E-15
RNF175	Ring finger protein 175	E3 ligase	2.9	2.89E-09
AOC2	Amine oxidase copper containing 2	Amine oxidases	2.8	1.1E-13
FMN1	Formin 1	Cytoskeleton	2.7	1.47E-25
MERTK	MER proto-oncogene, tyrosine kinase	Tyrosine kinase	2.7	3.97E-07
FKBP5	FKBP prolyl isomerase 5	Immunophilin protein family	2.7	2.04E-27
RGS2	Regulator of G protein signaling 2	G protein signaling	2.6	3.54E-09
SESN1	Sestrin 1	Sestrin family. P53	2.2	2.20E-19
CD163L1	CD163 molecule like 1	Scavenger receptor	2.7	6.01E-13
FPR1	Formyl peptide receptor 1	G-protein coupled receptor	2.6	1.89E-07
TFPI	Tissue factor pathway inhibitor	Serine protease inhibitor	2.3	3.54E-09
NES	Nestin	Intermediate filament protein family	−3.7	2.74E-12
METTL7B	Methyltransferase like 7B	Methyltransferase	−3.7	1.66E-10
TIE1	Tyrosine kinase with immunoglobulin like and EGF like domains 1	Protein tyrosine kinase	−3.7	1.66E-10
SNORD15B	Small nucleolar RNA, C/D box 15B	Small nucleolar RNAs	−3.7	4.78E-16
SNORA8	Small nucleolar RNA, H/ACA box 8	Small nucleolar RNAs	−3.4	2.04E-11
LGALS2	Galectin 2	Galactoside binding lectin	−3.4	3.58E-13
SNORD97	Small nucleolar RNA, C/D box 97	Small nucleolar RNAs	−2.9	2.04E-12
PRAM1	PML-RARA regulated adaptor molecule 1	Adaptor protein	−2.6	7.21E-25
COL6A1	Collagen type VI alpha 1 chain	Collagen (cytoskeleton)	−2.05	1.52E-06
SLC37A2	Solute carrier family 37 member 2	Solute carrier protein	−2.4	9.65E-07
SLC6A12	Solute carrier family 6 member 12	Solute carrier protein	−2.3	7.21E-15
SLC29A3	Solute carrier family 29 member 3	Solute carrier protein	−1.9	7.68E-13
CASS4	Cas scaffold protein family member 4	Scaffolding protein	−2.4	4.78E-08
UBTD1	Ubiquitin domain containing 1	E3 ligase	−1.7	1.87E-08
ITGB7	Integrin subunit beta 7	Integrin	−2.9	1.92E-11

Pathway enrichment analysis identified over 50 significant pathways affected by DEX treatment (Figure 1D). The most significant pathways were metabolic pathway (FDR = 4.1 × 10^−10^), lysosome (FDR = 6.3 × 10^−9^), phagosome (FDR = 3.9 × 10^−5^), FoxO signaling pathway (FDR = 7 × 10^−6^), transcriptional misregulation in cancer (FDR = 1.6 × 10^−6^), and regulation of actin cytoskeleton (FDR = 5.7 × 10^−5^).

### Effect of Dexamethasone on Metabolic Pathway

**Figure 2:**
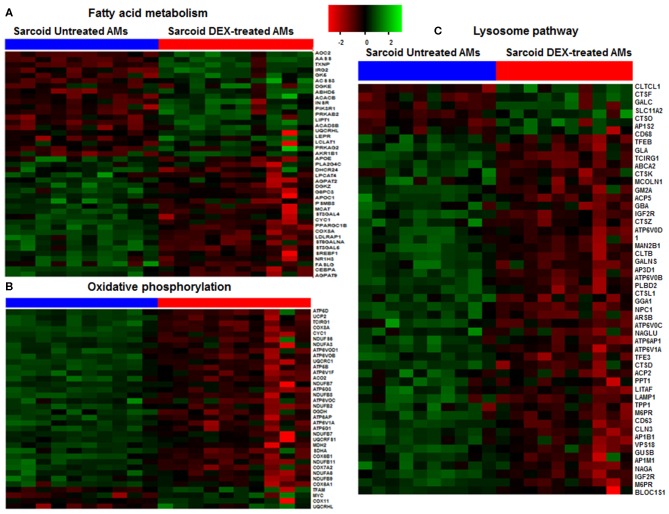
Heat map of DE genes involved in fatty acid metabolism, oxidative phosphorylation and lysosomal pathways. **(A)** Fatty acid metabolism, **(B)** Oxidative phosphorylation, **(C)** Lysosomal pathway. The thresholds used to select the DE genes: log_2_ FC > 0.6 and FDR < 5%. Green shade represents high expression and red shade represents low expression.

We found that 237 DE genes are involved in metabolic pathway of which 65 genes were upregulated and 172 genes were downregulated. To determine the metabolic pathways impacted by DEX treatment, we performed a pathway analysis of these 237 genes using Gene Trail 2. The most significant pathways were as following; oxidative phosphorylation (FDR = 1.4 × 10^−36^), carbon (FDR = 4.7 × 10^−34^), purine metabolism (FDR = 4.2 × 10^−22^), pyrimidine metabolism (FDR = 4.8 × 10^−18^), non-alcoholic fatty liver disease (NAFLD) (FDR = 2.3 × 10^−17^), glycolysis (FDR = 2.4 × 10^−16^), and fatty acid metabolism (FDR = 2.1 × 10^−12^). Figure 2 shows the heatmap of genes involved in fatty acid metabolism (A), oxidative phosphorylation (B), and lysosome pathway (C) in response to DEX-treatment. DEX-treatment led to downregulation of genes involved in glycolysis and oxidative phosphorylation. Specifically, DEX-treatment downregulated the expression of peroxisome proliferator-activated receptor γ (PPARγ) co-activator 1 Beta gene (PPARGC1B) (log_2_ FC = −1.4, FDR = 3.08 × 10^−9^). PPARγ co-activator is known to be the master regulator of mitochondrial biogenesis, oxidative phosphorylation and lipid metabolism (24, 25). In contrast, DEX increased expression of genes involved in amino sugar and nucleotide sugar metabolism. Fucose-1-phosphate guanylyltransferase (FPGT) increased in response to DEX treatment by 2 folds. Protein encoded by this gene participates in phosphorylation of L-fucose to form beta-L-fucose-1-phosphate which is a substrate of fucose-1-phosphate guanylyltransferase and GTP to form GDP-beta-L-fucose (26). AOC2 gene (log_2_ FC = 3, FDR = 1.1 × 10^−13^) catalyzes the oxidative conversion of amines to aldehydes and ammonia in the presence of copper and quinone cofactor and is associated with eye diseases (27). Glutamate-ammonia ligase (GLUL) expression is also increased in response to DEX (log_2_ FC = 2, FDR = 3.8 × 10^−11^). The protein encoded by this gene catalyzes the synthesis of glutamine from glutamate and ammonia in an ATP-dependent reaction (28). It is a key protein of glutamine synthesis and essential for cellular metabolism including macrophages (29). DEX upregulated the genes involved in insulin receptor signaling. For instance; insulin receptor substrate 2 (IRS2) (log_2_ FC = 1.2, FDR = 5.4 × 10^−5^), insulin receptor (INSR) (log_2_ FC = 1.0, FDR = 2.0 × 10^−3^), and phosphoinositide-3-kinase regulatory subunit 1 (PIK3R1) (log_2_ FC = 1.0, FDR = 3.4 × 10^−6^). AMP-activated protein kinase (AMPK) is an important energy-sensing enzyme that monitors cellular energy status. DEX upregulated the expression of genes that encode AMPK subunits, including catalytic subunit alpha 1 (PRKAA1) (log_2_ FC = 0.96, FDR = 1.8 × 10^−5^) and non-catalytic subunit beta 2 (PRKAB2) (log_2_ FC = 0.95, FDR = 6.2 × 10^−11^) (30). The catalytic subunit of AMPK protects cells from ATP depletion by switching off ATP-consuming biosynthetic pathways (30).

### Modulation of Phago-Lysosomal Genes by Dexamethasone

The lysosomal pathway was the second most impacted pathway in response to DEX treatment. Figure 2C shows the effect of DEX treatment on the gene expression involved in the lysosome pathway in sarcoidosis AMs. Among 44 lysosomal genes only 6 genes were upregulated while the rest were down regulated in response to DEX treatment. Clathrin heavy chain like (CLTCL) 1 expression increased 4 fold (log_2_ FC = 2.0, FDR = 8.2 × 10^−8^) in response to DEX treatment. Clathrin coated vesicles have been shown to endocytose accumulated protein aggregates and direct them to the lysosome for further degradation (31). Cathepsins represent the major components of the lysosomal proteolytic system. Cathepsin F (CTSF) (log_2_ FC = 1.5, FDR = 0.8 × 10^−7^) and cathepsin O (CTSO) (log_2_ FC = 0.65, FDR= 5.4 × 10^−3^) were upregulated. Galactosylceramidase (GALC) hydrolyzes the galactose ester bonds of galactosylceramide and was increased (log_2_ FC = 1.0, FDR = 9.1 × 10^−5^). Lysosomal related genes involved in V-ATPase dependent organelle acidification such as T Cell Immune Regulator 1 (TCIRG1) (log_2_ FC = −1.3, FDR = 1.4 × 10^−16^), lysosomal proteases (CTSK) (log_2_ FC = −1.1, FDR = 6.3 × 10^−4^), sulfatases (ARSB) (log_2_ FC = −0.8, FDR = 1.5 × 10^−5^), and lysosomal membrane proteins (LAMP) (log_2_ FC = −0.7, FDR = 3.5 × 10^−3^) were significantly decreased in response to DEX treatment. Cathepsin K (CTSK) expression was downregulated in sarcoid AMs after DEX treatment. CTSK, a lysosomal cysteine protease, is proteolytic for matrix proteins, including gelatin and fibrillar collagens. It has been shown to play a key role in protecting against lung fibrosis (28). The Mucolipin 1 (MCOLN1) gene that encodes a member of the transient receptor potential (TRP) cation channel gene family was downregulated (log_2_ FC = −1.1, FDR = 2.6 × 10^−10^). MCOLN1 has been suggested to regulate fusion/fission of vesicles in the endocytic pathway (32), and lysosomal ion homeostasis. Mutations in the MCOLN1 gene have been reported with lysosomal storage disease (33). The Transcription factor EB (TFEB) gene was downregulated (log_2_ FC = −1.7, FDR = 2.2 × 10^−9^). TFEB plays an important role in lysosomal biogenesis, autophagy and cellular metabolism (34). These data show that DEX treatment downregulates genes of the lysosomal pathway and lysosomal biogenesis that are involved in uptake and acidification processes and in the proteolysis of degraded proteins/debris in lysosomes.

In response to DEX most genes related to phagosome formation showed decreased expression, only 6 genes were upregulated. Upregulated genes in response to DEX treatment were as following: member RAS oncogene family RAB5A (log_2_ FC = 0.7, FDR = 3.39 × 10^−11^) that is required for the fusion of plasma membranes and early endosomes, TLR4 (log_2_ FC = 1.5, FDR = 2.8 × 10^−7^), TLR5 (log_2_ FC = 1.1, FDR = 4.1 × 10^−2^), TLR8 (log_2_ FC = 0.8, FDR = 4.1 × 10^−3^), dynein cytoplasmic 1 light intermediate chain 2 (DYNC1L) (log_2_ FC = 0.8, FDR = 5.7 × 10^−15^) and dynein cytoplasmic 2 heavy chain 1 (DYNC2H1) (log_2_ FC = 1.6, FDR = 1.3 × 10^−6^). This gene encodes a large cytoplasmic dynein protein involved in retrograde transport in the cilium and intra flagellar transport, a process required for ciliary/flagellar assembly. Cytoplasmic dynein-1 transports cargo into cell interior toward microtubule minus ends (35). Dynein-2, also known as intra flagellar transport dynein, moves cargoes along the axoneme of eukaryotic cilia and flagella (36). They are important for ciliary function, neuron and phagolysosome formation (30, 37). Aberrant function of the protein has been reported in ciliary dysfunction (35). Three genes encoding subunits of NADPH oxidase complex were downregulated. These were CYBA (p22-PHOX) (log_2_ FC = −0.7, FDR = 7.2 × 10^−9^), CYBB (log_2_ FC = −0.8, FDR = 1.0 × 10^−3^) and NCF2 (log_2_ FC = −0.9, FDR = 1.0 × 10^−4^). While NCF-1 gene expression was upregulated (log_2_ FC = 1.72, FDR = 3.7 × 10^−8^). NADPH oxidase is a key producer of reactive oxygen species (ROS) participating in killing of bacteria in phagosomes. Mutation in this gene is associated with chronic granulomatous disease (38).

### Transcriptional Misregulation in Cancer

DEX treatment of sarcoidosis AMs upregulated the expression of 18 genes and downregulated the expression of 20 genes related to transcriptional misregulation in cancer. The expression of 15-hydroxyprostaglandin dehydrogenase (HPGD) was increased log_2_ FC = 1.5, FDR = 9.3 × 10^−4^). HPGD is involved in the degradation of prostaglandin E2 (PGE_2_) and hence suppresses tumor formation and inhibits inflammation induced by PGE_2_ (39, 40). DEX treatment increased the expression of the FLT3 gene (log_2_ FC = 1.5, FDR = 1.4 × 10^−3^). This gene encodes a receptor tyrosine kinase promoting Ras signaling, which plays a key role in survival, proliferation and differentiation of hematopoietic cells (41–43). Myocyte enhancer factor 2C (MEF2C) expression was upregulated (log_2_ FC = 1.0 × 10^−6^, FDR = 3.8 × 10^−8^). MEF2C, a member of the MADS family of transcription factors, regulates hematopoietic self-renewal and differentiation, supports the proliferation of leukemias, and is associated with increased risk of relapse in leukemia patients as its phosphorylation leads to chemotherapy resistance (44). The NFKB inhibitor zeta (NFKBIZ) gene that encodes for IkappaB-zeta, a transcriptional regulator for NF-kappaB, was upregulated (log_2_ FC = 1.5, FDR = 1.0 × 10^−5^) with DEX treatment. It has been shown to induce pro-inflammatory responses and lymphoproliferative disorders and in solid tumors (45). The MDM2 proto-oncogene (log_2_ FC = 1.5, FDR = 1.0 × 10^−6^) encodes a nuclear-localized E3 ubiquitin ligase, promoting tumor formation by targeting tumor suppressor proteins, such as p53, for proteasomal degradation. The upregulated expression of the FLT3, MEF2C, NFKBIZ, and MDM2 genes in sarcoid AMs suggests the potential side effect of DEX and it may lead to increased risk of tumor cell proliferation. DEX treatment modulated expression of several genes related to focal adhesion and cytoskeleton, including integrin families. Cyclin D2 (CCND2) functions as regulators of CDK kinases important for cell cycle G1/S transition was downregulated (log_2_ FC = −1.7, FDR = 1.3 × 10^−4^) (46).

### Regulation of Actin Cytoskeleton

One of the major pathways affected by DEX treatment was regulation of the actin cytoskeleton. Actins are highly conserved proteins that are involved in cell motility, structure, integrity, and intercellular signaling (47). Plakophilin 2 (PKP2) encoding a plakophilin protein, was significantly upregulated (log_2_ log_2_ FC = 4.6, FDR = 7.9 × 10^−19^). Plakophilin 2 participates in linking cadherins to intermediate filaments in the cytoskeleton and cardiac muscles. It has been shown to be associated with several types of cancers in humans (48). PKP2 regulates calcium signaling in cardiac muscles (49). The slingshot homolog 2 (SSH2) gene was upregulated (log_2_ FC = 1.2, FDR = 1.0 × 10^−6^). SSH2 encodes a protein tyrosine phosphatase, which regulates actin filaments by dephosphorylating and activating cofilin which promotes actin filament depolymerization (50). The actin regulatory protein, cofilin/actin depolymerization factor (ADF), serves a vital function in cells by severing filaments, thereby increasing the number of filament ends from which polymerization and depolymerization can occur (47). The cofilin pathway is important for actin dynamics, cell movement, cytoskeleton, distribution of receptors and synapse formation and migration of immune cells. It is critical for phagocytosis, motility, and antigen presentation in macrophages, dendritic cells, and neuronal cells (51). Similarly, we found that DEX treatment downregulated the expression of several integrin membrane protein genes. Few of these genes are: Integrin subunit beta and alpha, ITGB7 and ITGA7 (log_2_ FC = −1.2, FDR = 4.6 × 10^−5^), ITGAM, ITGA6, ITGB2. These genes code for integrins/adhesion receptors involved in leucocyte migration.

### Effect of Dexamethasone on Genes Related to Inflammation

The TSC22 domain family member 3 gene (TSC22D3) was found to be highly upregulated in sarcoid AMs after DEX treatment (Table 2). TSC22D3 gene encodes GC-induced leucine zipper transcription factor, whose expression is rapidly induced by GC, IL-10 and TGF-β (52, 53). This protein has been shown to play a key role in immunosuppressive effects of GCs. DEX upregulated the expression of genes involved in the negative regulation of inflammation, including Dual specificity phosphatase (DUSP)1, also known as MKP1, which is a phosphatase involved in the dephosphorylation of MAPKs (15), and suppressor of cytokine signaling (SOCS4 and SOCS6). DEX downregulated the expression of various genes that play a key role in inflammation. Gene expression for interleukin 6 receptor complex (IL6R), TNF-receptor superfamily (TNFRSF4) and MyD88, essential signaling molecules in TLR and IL-1 signaling were decreased in response to dexamethasone treatment.

### Effect of Dexamethasone on Transcriptional Signature of Sarcoidosis CD14^+^ Monocytes

**Figure 3:**
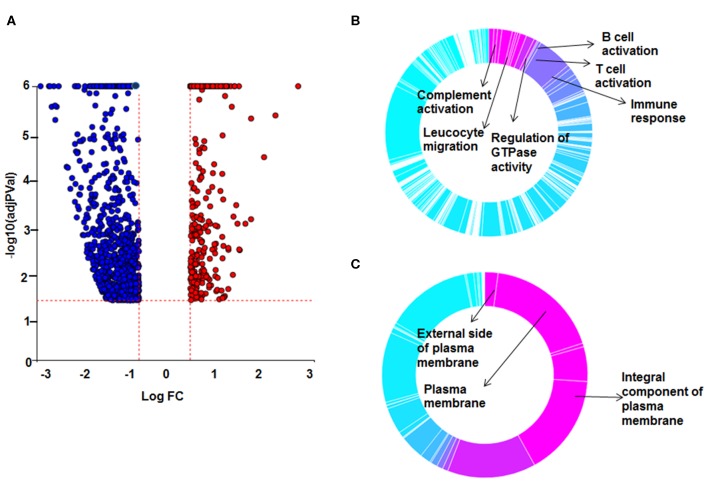
Differential gene expression and Gene Ontology (GO) processes between sarcoid DEX-treated and untreated sarcoid monocytes. **(A)** Volcano plot: All 1,958 significant DE genes analyzed by DEseq2 analysis tool are represented in terms of their measured expression fold change (log_2_FC) on x-axis and the significance of the change (–log_10_ adj *p*-value) on y-axis. The dotted lines represent the thresholds used to select the DE genes: log_2_ FC > 0.6 and significance FDR<5%. The up-regulated genes (positive log_2_FC) are shown in red, while the down-regulated (negative log_2_FC) genes are shown in blue. **(B,C)** Gene Ontology (GO) analysis of DE genes was done using ipathwayGuide analysis tool. To assess the enrichment of GO terms Elim pruning method was used. GO analysis identified top **(B)** biological processes and **(C)** cellular components.

**Figure 4:**
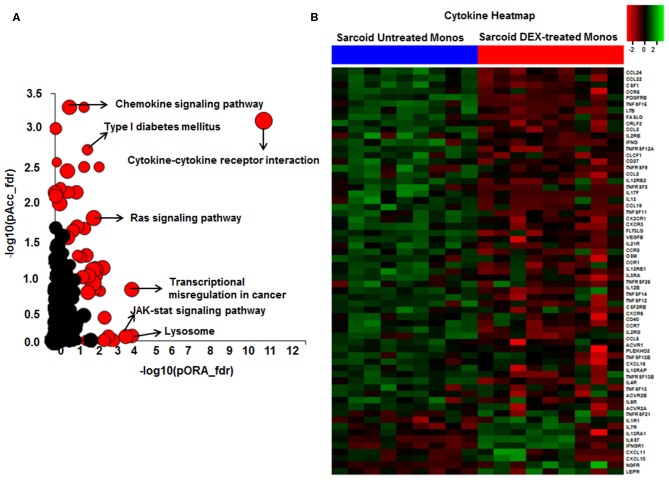
Significant pathways enriched in DEX-treated compared to untreated sarcoid monocytes. **(A)** Pathway analysis was done on the DE genes (log_2_FC > 0.6 and FDR < 5%) using iPathwayGuide analysis tool that uses two types of evidence: the over-representation on the horizontal axis (pORA) and the perturbation on the vertical axis (pAcc). Significant pathways (FDR < 5%) are shown in red, whereas non-significant are in black. The size of the circle is proportional to the number of genes in that pathway. **(B)** Heat map of DE genes involved in cytokine-cytokine receptor interaction pathway (log_2_ FC > 0.6 with FDR < 5%). Green shade represents high expression and red shade represents low expression.

To decrease the effect of genetic variation, we used the same cohort of patients used for the AMs study. To identify genes responsive to GC on sarcoidosis monocytes, we isolated CD14^+^ monocytes from PBMCs and treated in a similar fashion with DEX. In response to DEX treatment 1,958 genes were differentially expressed in monocytes (log_2_-fold change of 0.6 and FDR < 5%) (Figure 3A). The Gene ontology (GO) enrichment analysis showed that the significant biological processes impacted after DEX treatment were classic complement activation, leukocyte, T and B cell activation as well as phagocytosis (Figure 3B) and significant cellular components impacted are shown in Figure 3C. Next, we performed pathway enrichment analyses. The top impacted pathways in monocytes were as following: cytokine-cytokine receptor interaction (FDR = 6 × 10^−6^), transcriptional misregulation in cancer (FDR = 1 × 10^−4^), Type I DM (FDR = 2.6 × 10^−3^), cell adhesion (FDR = 0.003), cytoskeleton (FDR = 2.0 × 10^−3^), RAS signaling (FDR = 2.0 × 10^−3^), and JAK-STAT pathway (FDR = 2.0 × 10^−3^) (Figure 4A). The top most impacted pathway in CD14^+^ monocytes in response to DEX treatment was related to cytokine-cytokine receptor interaction. DEX treatment decreased expression of numerous cytokine and chemokine genes in sarcoidosis monocytes, while increasing the expression of CXCL10 and IL-10. In contrast, DEX treatment led to a significant decrease in expression of CCL22 (also known as macrophage-derived chemokine) and CCL24. CCL22 is produced by myeloid cells and regulates migration of leukocytes (54). Many genes related to tumor necrosis factor alpha superfamily members, *TNFRSF8, 9, 11, 14, and 15* were down regulated in response to DEX treatment. Figure 4B shows the heatmap of genes involved in cytokine pathways. Similarly, gene expression for several TNF receptor associated factors (TRAFs) that are important for the TLR and TNF signaling activation were decreased. In contrast, DEX treatment led to increased expression of negative regulators of TLR signaling, TNF alpha induced protein 3 (TNFAIP3-A20). Similar to AMs, the pathway related to transcription misregulation in cancer was significantly impacted in response to DEX treatment of sarcoidosis monocytes.

The top most upregulated gene in response to DEX treatment in sarcoidosis monocytes was actin binding LIM protein family member 3 (ABLIM3) (log_2_ FC = 3.1, FDR = 1.0 × 10^−6^) (Table 3). The encoded protein interacts with F actin filaments and is a component of adherents junctions in several cell types, including cardiac myocytes (55). Several genes related to retinoid X receptors (RXRs) and retinoic acid receptors (RARs) were downregulated in response to DEX treatment. These are important members of the steroid/thyroid hormone receptors acting predominantly as transcription factors (56).

**Table 3:** 10 top up and down-regulated genes in monocytes in response to dexamethasone treatment.

**Gene symbol**	**Gene name**	**Type**	**Log fold change**	**FDR *p*-value**
ABLIM3	Actin binding LIM protein family member 3	Cytoskeleton	3.1	5.89E-09
SERPINE1	Serpin family E member 1	Serine protease	2.4	1.0E-06
MT1G	Metallothionein 1G		2.5	4.37E-06
CD163L1	CD163 molecule like 1		1.9	1.0E-06
FPGT-TNNI3K	FPGT-TNNI3K	Cytoskeleton	2.7	1.0E-06
SLC6A13	Solute carrier family 6 member 13	Solute carrier protein	2.7	1.0E-06
FKBP5	FKBP prolyl isomerase 5	Immunophilin protein family	2.05	5.11E-06
RGS2	Regulator of G protein signaling 2	G protein signaling	2.4	2.82E-06
HSPE1P18	Heat Shock 10kDa Protein 1 Pseudogene 18	Heat shock protein	2.2	1.0E-06
TUBE1	Tubulin epsilon 1	Cytoskeleton	2.3	1.64E-06
RYR2	Ryanodine receptor 2	ER resident	2.7	3.59E-05
CCL24	C-C motif chemokine ligand 24	Cytokine	−3.0	3.70E-08
CCL22	C-C motif chemokine ligand 22	Cytokine	−3.7	3.70E-08
FPR3	Formyl peptide receptor 3	G-protein-coupled receptors	−3.4	3.25E-09
RAPGEF5	Rap guanine nucleotide exchange factor 5	Ras family member	−2.6	2.12E-04
HSD3B7	Hydroxy-delta-5-steroid dehydrogenase	Member of the short-chain dehydrogenase/reductase superfamily	−2.05	2.65E-06
FEZ1	Fasciculation and elongation protein zeta 1	Zygin family	−2.4	9.40E-07
ADAMTS14	ADAM metallopeptidase with thrombospondin type 1 motif 14	Metallopeptidase	−1.9	2.9E-03
KCNN4	Potassium calcium-activated channel subfamily N member 4	Voltage-independent potassium channel	−2.4	1.20E-06
ATP8B4	ATPase phospholipid transporting 8B4	Phospholipid transport	−1.7	1.43E-06
IL32	Interleukin 32	Cytokine	−1.8	1.43E-06

### Concordance Between DEX-Treated Sarcoidosis AMs and Monocytes

**Figure 5:**
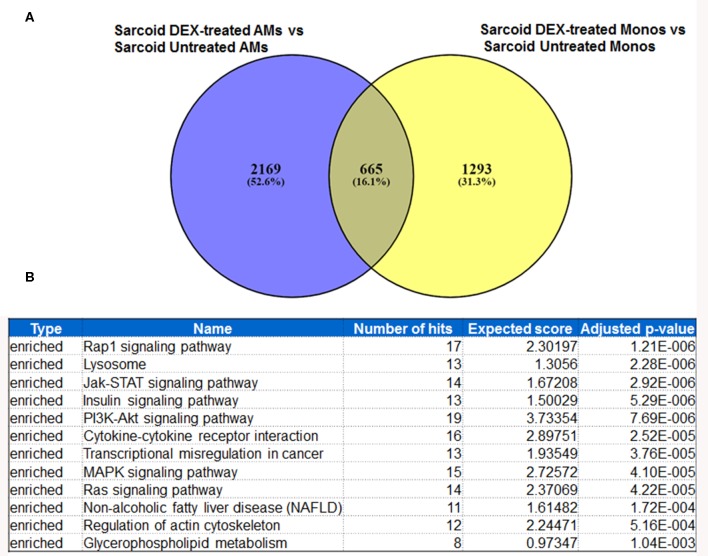
Metanalysis of DE genes between two groups: DEX-treated vs. untreated sarcoid AMs and DEX-treated vs. untreated sarcoid monocytes. **(A)** Venn diagram of DE genes showing 2,169 DE genes exclusively expressed in DEX-treated sarcoid AMs whereas 1293 DE genes were exclusively expressed in DEX-treated sarcoid monocytes. 665 DE genes overlap between the two groups **(B)** GeneTrail pathway analysis of common 665 DE genes between DEX treated sarcoid AMs and DEX treated sarcoid monocytes shows top 12 enriched significant pathways.

**Figure 6:**
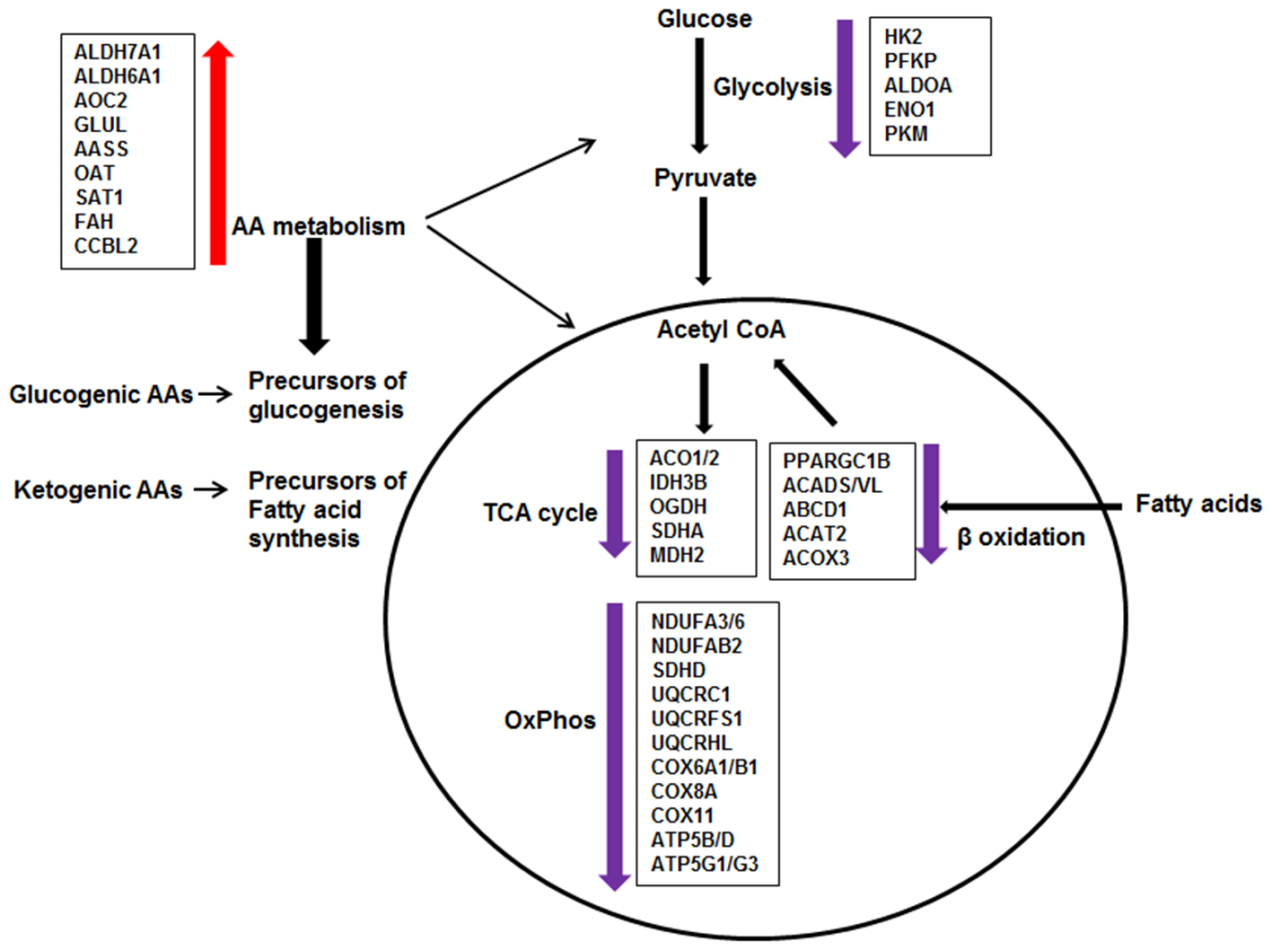
Effect of DEX on metabolic pathways in sarcoid AMs. The pathway analysis of metabolic DE genes showed that the major pathways affected in DEX-treated sarcoid AMs as compared to untreated sarcoid AMS were: Glycolysis, Citric acid cycle (TCA), Oxidative phosphorylation (OxPhos), β-oxidation of fatty acids and Amino acid (AA) metabolism. DEX-treatment resulted in the downregulation of genes involved in glycolysis, TCA cycle, Oxphos and β-oxidation of fatty acids. Alteration in these pathways suggests that glucose metabolism is decreased; glucose is not being utilized completely to generate energy producing molecules ATP and NADH. Reduced β-oxidation of fatty acids suggests that fatty acids are not being transported and degraded optimally in mitochondria to generate energy. Suboptimal fatty acid oxidation may result in the accumulation of long chain fatty acids and metabolites. The upregulation of genes involved in AA metabolism shows that DEX-treated sarcoid AMs may switch to AA catabolism as a source of energy. AAs catabolism results in the conversion of AAs either to pyruvate or intermediates of TCA cycle for energy production or generation of precursors of glucogenesis and fatty acid synthesis.

In response to DEX 2169 genes were exclusively differentially expressed in sarcoid AMs, while 1293 DE genes were found in sarcoid monocytes as shown in Venn diagram (Figure 5A). These results show that the impact of DEX treatment is more profound on sarcoid AMs as compared to monocytes. Figure 6 shows 665 DE genes overlapping in DEX-treated sarcoidosis AMs and monocytes. We found that the effect of DEX on overlap DE genes was similar except for a few genes. DEX treatment upregulated the expression of activin A receptor type 2B (ACVR2B), MYC proto-oncogene, bHLH transcription factor (MYC), zinc finger and BTB domain containing 18 (ZBTB18) genes in AMs whereas it downregulated their expression in monocytes. ZBTB18 encodes a C2H2-type zinc finger protein and acts a transcriptional repressor of genes involved in neuronal development. Further, we performed the pathway enrichment analysis on the common DE genes in both cell types. The common pathways that were enriched in both DEX-treated AMs and monocytes were Rap1 signaling pathway, lysosomal pathway, PI3kinase signaling pathway, transcriptional misregulation in cancer, Type I diabetes mellitus (Figure 5B).

## Discussion

Macrophages have a central role in the maintenance of immunological homeostasis and host-defense. They play a crucial role in granuloma formation and provide metabolic cues for T cell responses in sarcoidosis. Alveolar macrophages, similar to other tissue associated macrophages have evolved to perform phagocytic clearance of pathogens and dying cells in the lungs immune-surveillance of the lung for inhaled pathogens as well as homeostatic regulation of lung tissues (11, 57). Circulating monocytes have the potential to differentiate into monocyte derived tissue macrophages and to migrate and interact with antigen-specific T and B lymphocytes to initiate adaptive immune responses.

Glucocorticoids modulate host immune responses to pathogens. In inflammatory diseases, including sarcoidosis, GCs have been used as an immunosuppressive drug to reduce inflammation (58). It has been shown that GCs suppress the expression of inflammatory genes, including TNF-α and IFN-γ, and chemokines that are important for the granuloma formation and cell mediated Th1 responses. GCs are the only FDA approved drug to treat pulmonary and extrapulmonary sarcoidosis (59). However, despite their widespread use, there is a lack of evidence for their long term survival benefit in sarcoidosis or prevention of end organ damage such as lung fibrosis (58). In contrast, long term use of GCs has been shown to have significant side effects affecting all systems in the body (60). The most common side effects are excessive weight gain, insomnia, diabetes, osteoporosis, arterial hypertension and depression (60).

**Figure 7:**
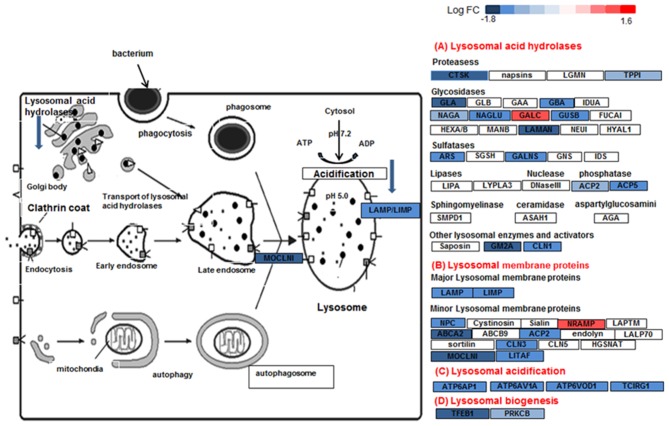
Lysosome pathway in DEX-treated sarcoid AMs. Graphic illustration of pathway analysis of DE (log_2_ FC > 0.6 with FDR < 0.05) genes related to lysosome pathway in sarcoid-DEX treated AMs. The pathway diagram is overlaid with the computed perturbation of each gene. The perturbation accounts both for the gene's measured fold changes and for accumulated perturbation propagated from any upstream genes (accumulation). The color intensity corresponds to the level of upregulation (red) or downregulation (blue) of the DE genes in sarcoid-DEX treated AMs vs. sarcoid untreated AMs.

To determine the effect of GCs on transcriptome profiling of sarcoid AMs and CD14^+^ monocytes, we compared the RNA-seq data of sarcoidosis AMs and CD14^+^ cells treated with dexamethasone *in vitro* vs. untreated sarcoidosis AMs and CD14^+^ monocytes, respectively. To decrease the confounding factors related to human genetic variation, we isolated the CD14^+^ peripheral blood monocytes and AMs from the BALs of the same sarcoidosis subjects and compared pairwise RNA seq results. We identified two major pathways impacted after DEX treatment: metabolic and phagolysosomal pathways. Metabolic reprogramming in macrophages is critically important for their effector function (61, 62). Figure 7 summarizes perturbation of genes related to metabolic pathways. We found that DEX treatment leads to suppression of gene expression related to glycolysis, fatty acid oxidation, TCA cycle and oxidative phosphorylation (Figure 6). In contrast, GCs enhance the expression of genes related to amino acid degradation pathways and generation of glutamine from glutamate (GLUL). Dexamethasone effected genes involved in metabolism of glucogenic amino acids (AAs) and ketogenic AAs, suggesting increased glucogenesis and fatty acid precursors (Figure 6). These alterations in response to dexamethasone explains some of the adverse effects seen in these patients after treatment with GCs, including loss of muscle mass, and lipodystrophy (63).

Other important dexamethasone effects were its effects on genes involved in lysosome and phagosome functions. Lysosomes are the common platform for endocytosis, phagocytosis, autophagy and biosynthetic routes. They receive both extracellular and intracellular-derived molecular cargo, damaged organelles, engulfed dead cells, and foreign particulates like bacteria for digestion. Tissue macrophages, including AMs, are sentinel cells that are equipped with a series of mechanisms of vesicular trafficking to orchestrate the elimination of bacteria as well as dead cells and debris (60). Clearance of bacteria by macrophages involves internalization of the microorganisms into phagosomes, which are then delivered to endo-lysosomes for enzymatic degradation. Lysosomes are a central organelle in the processing of exogenous and intracellular biomolecules. Thus, the final clearance of pathogens depend on lysosomal function (64). Lysosomes integrate and facilitate cross-talk between nutritional signals such as AA and energy levels, membrane damage and infection, and ultimately enable responses such as autophagy, cell growth, membrane repair and microbe clearance (65, 66). Lysosomes are hubs for cellular signaling and nutritional sensing through interplay with mTOR, AMPK, and GSK signaling (65). DEX-treated AMs show significantly decreased gene expression for the phagosome and lysosome machinery. For instance, transcription factor EB (TFEB), a master regulator of lysosomal biogenesis and fusion (65) was significantly downregulated in sarcoidosis AMs in response to DEX. Figure 7 shows the downregulation of lysosome pathway genes encoding lysosomal acid hydrolases, membrane proteins, acidification and biogenesis proteins. Our results show that DEX-treatment led to the downregulation of two important genes, TFEB and PPARGC1 (PGC1) that play an important role in lysosomal and mitochondrial biogenesis and cellular metabolism. TFEB has been shown to control lipid catabolism and control the expression of PGC1α (34, 67). The DEX induced downregulation of TFEB, PGC1α, mitochondrial Acyl-CoA Dehydrogenase short chain fatty acid (ACADS) and very long-chain acyl-CoA dehydrogenase (VLCAD), as well as lysosomal genes in sarcoid AMs. This suggests that DEX-treatment decreases fatty acid oxidation and thus may lead to increased lipid deposition. Furthermore, DEX-treatment upregulated the expression of the CLTC1 gene (lysosomal pathway) that may contribute to accumulation of clathrin coated vesicles containing aggregated proteins. In contrast, the downregulation of lysosomal genes involved in proteolysis and acidification suggests that DEX-treatment inhibits the proteolytic degradation. This may result in lysosomal overload with protein aggregates and fatty acid deposits. Decreased lysosomal function may lead to an increase in unfolded protein accumulation and finally to unfolded protein response and ER stress (68). Because pathogens and antigens may play a role in the pathogenesis of sarcoidosis, the downregulation of lysosomes and phagosome by DEX may have negative implications for the disease progression.

Similar to rheumatoid arthritis, some studies have shown an increased risk of developing cancer in sarcoidosis patients, while others could not confirm such association (69–73). While it is difficult to interpret uncontrolled and retrospective observational studies, the increased risk is partly attributed to uncontrolled inflammation. Our study showed that transcriptional misregulation in cancer was also one of the enriched pathways in response to DEX treatment. We found that DEX treatment upregulated the expression of numerous genes involved in cell proliferation, tumor formation and lymphoproliferative disorders. Interestingly, DEX treatment led to upregulation of this pathway in the monocytes of sarcoidosis subjects, suggesting that this effect is a global effect. These results demonstrate that long-term usage of DEX may lead to uncontrolled proliferation. For example: RAS superfamily gene are proto-oncogene that are involved in the development of various cancers (74, 75). It has been shown that DEX upregulates several members of this family (76). Our data is in line with previous RNA seq data of cultured human pulmonary type II A549 cells showing upregulation of transcriptional misregulation in cancer in response to corticosteroids (77).

In response to DEX treatment, we identified derangement of large numbers of genes regulating the cytoskeleton. Most genes belong to integrin family, but also genes involved in desmosomes such as PKP2 gene encoding a protein which links cadherins to intermediate filaments in the cytoskeleton. Aberrant regulation of these genes, including PKP2, ITGA6, and seven are important for cytoskeleton and cell-cell and cell-matrix interactions and are associated with cardiomyopathy, arrhythmogenic R ventricular cardiomyopathy and hypertrophic cardiomyopathy. Collectively, these data suggest that dexamethasone drives a broad gene expression program promoting matrix stiffening and supporting fibroblast proliferation and selective but coordinated suppression of genes encoding collagen-degrading enzymes.

On the other hand, our results show that DEX treatment altered the expression of genes involved in inflammation and immune responses. DEX treatment led to decreased expression of genes related to inflammation including IL6R, IL-17R, TNFRSF4, and MyD88. Along with this DEX treatment upregulated the expression of several genes that suppress inflammation, including Glucocorticoid-Induced Leucine Zipper Protein (GLIZ, also known as TSC22D3), dual specificity phosphatase 1 (DUSP1, also known as MKP-1), Suppressor of Cytokine Signaling (SOCS) 4 and 6. We have previously shown that DUSP1 expression is decreased in sarcoidosis AMs and monocytes and GCs induced DUSP1 expression at the protein level (15). Current RNA seq data in an independent cohort of patients is in agreement with our previous results.

## Concluding Remarks

For more than 60 years GCs have been used as anti-inflammatory drugs to treat a variety of inflammatory diseases such as rheumatoid arthritis and pulmonary diseases such as sarcoidosis. Given the fact that macrophages play a critical role in infectious and non-infectious granulomatous diseases (78, 79), it is of surprise that the effects of GCs on macrophages are less well-documented. Here we show that DEX treatment led to profound transcriptomic changes related to cellular metabolisms, lysosomes/phagosomes, and cytoskeleton in lung tissue macrophages. While similar effects were observed after DEX treatment on isolated CD14^+^ monocytes of the same subjects, the effect on cytokine-cytokine receptor interaction was more prominent in monocytes, suggesting that the effect on lung tissue macrophages are more likely due to lysosomal function metabolic derangement. Further studies need to determine the effects of DEX on proteome profile in different cell types and correlation with RNA seq data.

## Data Availability Statement

The datasets generated for this study can be found in the NCBI BioProject database with accession number PRJNA630000.

## Ethics Statement

The studies involving human participants were reviewed and approved by the Committee for Investigations Involving Human Subjects at Wayne State University. The IRB number for this study is 055208MP4E. The patients/participants provided their written informed consent to participate in this study.

## Author Contributions

JT conducted the experiments, analyzed the results, and she contributed in writing the manuscript. CB critically reviewed the manuscript. ES performed the statistical analysis of the RNA-seq data. FL and RP-R reviewed the data analysis and the manuscript. LS conceived and designed the study, participated in all areas of the research such as patients' selection and oversaw patient enrollment, data analysis, and writing of the manuscript.

## Conflict of Interest

The authors declare that the research was conducted in the absence of any commercial or financial relationships that could be construed as a potential conflict of interest.
