# Supplementary material for: Derangement of Metabolic and Lysosomal Gene Profiles in Response to Dexamethasone Treatment in Sarcoidosis
Source: Front Immunol. 2020 May 12;11:779. doi: 10.3389/fimmu.2020.00779 (PMC7235403; doi:10.3389/fimmu.2020.00779)
Supplement: Supplementary file 1 [file Data_Sheet_1.docx]

**Figure S1**


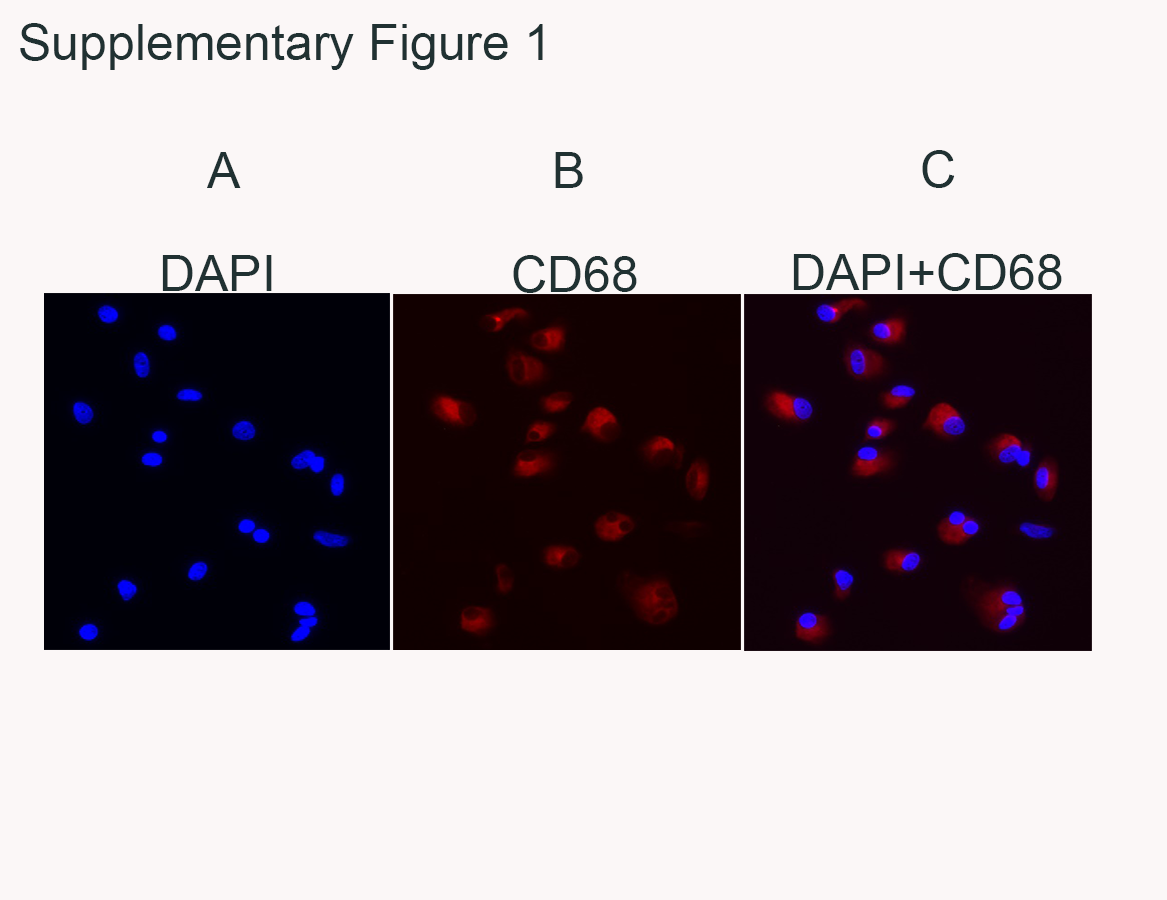


**Fig.S1.**  **Immunostaining of AMs.** AMs (1x 10^5^) were allowed to adhere overnight on chamber slides. Next day the cells were washed and fixed with 4% paraformaldehyde for 20 minutes at room temperature (RT). After fixation cells were permeabilized with 0.1% TritonX-100, blocked with 2%BSA and then immunostained with CD68 antibody (Santa Cruz) overnight at 4^0^C. The cells were washed and stained with AF568-conjugated secondary antibody for 1h at RT. The cells were washed three times with PBS for 5 min, the slide was mounted with a drop of ProLong Gold antifade reagent with DAPI (Invitrogen). The slide was examined using an immunofluorescent microscope ((AX10, Zeiss). Staining of sarcoid AMs (A) nuclear staining with DAPI (B) staining with CD68 (C) merged image stained with CD68 and DAPI showing > 99% of cells are CD68-positive AMs.
